# Influence of a Standardized Lunging Exercise Test on BALF Cytology in Horses Suffering from Mild–Moderate Equine Asthma †

**DOI:** 10.3390/ani15162428

**Published:** 2025-08-19

**Authors:** Lioba Lendl, Caroline Wirth, Roswitha Merle, Ann Kristin Barton

**Affiliations:** 1 Equine Clinic Hochmoor, 48712 Gescher, Germany; 2 School of Veterinary Medicine, Freie Universitaet Berlin, 14163 Berlin, Germany; 3 Institute of Veterinary Epidemiology and Biostatistics, School of Veterinary Medicine, Freie Universitaet Berlin, 14163 Berlin, Germany

**Keywords:** equine asthma, mild–moderate equine asthma, exercise test, diagnosis

## Abstract

Mild–moderate equine asthma (MEA) is a very common but underdiagnosed pulmonary disease in horses, with mild cases not showing clinical respiratory signs. This study evaluates the influence of a standardized lunging exercise test (SLET) on bronchoalveolar lavage fluid (BALF) cytology in MEA horses. We hypothesized that SLET would increase the total nucleated cell count (TNCC) and/or percentages of inflammatory cells associated with EA. In a prospective, randomized, non-blinded, between-subjects study design of two independent groups, 39 horses (17 mild and 22 moderate) were included. They were chosen out of a cohort of horses undergoing respiratory investigations (history, clinical examination, and clinical pathology (white blood cells (WBC) and arterial blood gas analysis (aBGA)) consistent with MEA, using a scoring system in a clinical setting of an equine referral clinic. Bronchoalveolar lavage (BAL) was performed 30 min post-SLET in 16 randomly chosen horses. The other horses underwent BAL without SLET. The SLET resulted in a statistically significant increase (*p* < 0.001) in the proportions of neutrophils in BALF cytology, and in an increased chance of confirmation of the presumed diagnosis in horses with mild phenotypes (*p* < 0.001, OR = 8.00, CI = 1.28–50.04), while moderate phenotypes were less frequently diagnosed. Exercise had no association with cytology across all horses. Unexpectedly, the SLET group of horses with a moderate phenotype had a statistically significant lower TNCC (*p* = 0.035). In conclusion, an SLET prior to BAL might increase the chance of an MEA diagnosis.

## Simple Summary

Equine asthma is a common pulmonary disorder, which is classified into mild–moderate and severe types. Mild–moderate equine asthma (MEA) is commonly underdiagnosed due to a lack of clinical signs or subtle presentation, such as poor performance. MEA may progress into the severe type. An early diagnosis might prevent clinical deterioration of the disease. This study evaluated the effects of a standardized lunging exercise test prior to bronchoalveolar lavage on the results of cytology in 39 MEA horses. In equine practice, the evaluation of cells in the bronchoalveolar lavage fluid is a very important part of diagnostics, as it allows one to classify and grade the inflammation in the small airways of the lung and is more widely available than lung function tests. Lunging exercise resulted in an increase in the proportions of neutrophils—which are the most common inflammatory cells in equine asthma—in bronchoalveolar lavage fluid (*p* < 0.001), and in an increased chance of confirmation of the presumed diagnosis, which was statistically significant (*p* < 0.001) in mildly affected horses only. There was no significant effect when comparing it to pre-lunging values of the entire group of horses. Therefore, a lunging exercise test might be a practical tool to identify mild cases at an early stage.

## 1. Introduction

Equine asthma is a common inflammatory disease of the lower airways in horses, usually neutrophilic, and may be associated with respiratory clinical signs or a loss of performance only. In horses, two phenotypes have been determined: a mild-to-moderate form (MEA) and a severe form (SEA) [1]. With a high prevalence of up to 70%, depending on study population and design, MEA is more frequently diagnosed in young horses under the age of 7 [2,3,4]. SEA is a chronic and progressive disease and is more prevalent in older horses, with a global prevalence of up to 14% [5]. In contrast to horses suffering from SEA, most of the horses with MEA appear to recover [1], as irreversible remodeling has not yet occurred. The probability of recovery is contingent on the extent of EA characteristics such as dyscrinia, hyperplasia of the goblet cells, and obstruction caused by mass hypertrophy of the smooth bronchial musculature [1]. In MEA, low-grade respiratory symptoms and a slight decrease in performance may be the only indicators of disease. At the Havemeyer Workshop 2019, a recommendation was made for further subdivision into mild and moderate phenotypes. Horses with EA exhibiting substandard performance yet lacking clinical indicators of respiratory disease may be designated as the mild form. Conversely, horses manifesting respiratory clinical signs would be classified as the moderate form. As a result of bronchoconstriction, horses with SEA in exacerbation show an elevated respiratory rate, abdominal breathing, and dyspnea at rest. One study indicated that the severe form of equine asthma can develop from the mild-to-moderate form [6], which is supported in an expert-opinion consensus paper [7]. Due to the limited research, it is unclear whether the mild-to-moderate form is actually developing into a severe form. Alternatively, due to the high prevalence of MEA, it is possible that some of these horses in the studies are developing symptoms that are incorrectly diagnosed as SEA. In horses suffering from SEA, progression of the disease leads to remodeling of the bronchial smooth muscle, the pulmonary arteries, and the extracellular matrix of the lamina propria. Remodeling of the bronchial smooth muscle is not fully reversible. Horses with SEA in remission exhibit a 50% increase in airway smooth muscle (ASM) compared to healthy horses [8]. In contrast, remodeling of the pulmonary arteries and extracellular matrix appears to be largely reversible after environmental improvement and inhalation therapy [9,10]. It is therefore recommended to achieve diagnosis early on in the disease process, as even horses suffering from MEA may already demonstrate remodeling with epithelial hyperplasia, a thickened lamina propria, and smooth muscle fibrosis [11]. The prognosis for recovery is dependent upon timely diagnosis of MEA, which is only possible before irreversible remodeling occurs. In the clinical setting it is frequently difficult to distinguish between mild asthmatics and healthy horses, but this is important for the therapeutic intervention. To date, EA is an incurable disease. Therefore, appropriate management is important for all horses, but particularly for those in which remodeling has occurred, to avoid further exacerbations and hopefully slow the progression of the disease. Given such a high prevalence, early and accurate diagnosis of MEA is therefore important. Unfortunately, a significant proportion of individuals affected by MEA probably remain undiagnosed, primarily due to absent or subtle clinical signs. There is a compelling need for heightened awareness of EA as a possible diagnosis in cases presenting with poor performance.

The latest has research identified multiple factors, such as short-term exercise, cold air, and exercise-induced pulmonary hemorrhage, that influence the cytology of the lower airways in the short term, which could be crucial for diagnostics [12,13,14,15,16]. Some might be helpful for an early diagnosis of MEA, as BALF cytology may be unremarkable in subclinical cases and low-grade disease. A short-term exercise test prior to bronchoscopy with BAL has been suggested to be a practical tool in clinical routine [13]. An exercise test enabled an EA diagnosis in asymptomatic racehorses due to an increased total nucleated cell count [12]. Another study found a negative effect on respiratory rate recovery after a submaximal lunging test in mild–moderate equine asthmatics, suggesting that an exercise test may be a viable diagnostic tool for mild–moderate asthmatics [17].

The present study investigated the association of exercise and BALF cytology in horses with MEA [18]. Unfortunately, lung function tests (LFTs) are not widely available in equine practice, despite being the reference standard. In a clinical setting, scoring systems including clinical examination, arterial blood gas analysis, and BALF cytology are commonly used. Even when using scoring systems including multiple parameters, MEA may remain undiagnosed, as BALF cytology can be unremarkable in remission, showing few or no clinical signs, with no signs of bronchoconstriction in aBGA, and LFTs may not be available. This was the background of the present study and the reason why we tested SLETs as a possible method to increase the chance of cytological diagnosis. We hypothesized that BALF cytology of exercise-stressed asthmatic horses would allow for a more definitive cytological diagnosis due to an increased total nucleated cell count (TNCC) or increased percentages of neutrophils, eosinophils, mast cells, lymphocytes, or macrophages.

## 2. Materials and Methods

### 2.1. Animals

This prospective clinical study included 45 horses of various ages (from 4 to 23 years), sexes (mares: 21; geldings: 22; stallions: 2), heights (from 146 cm to 179 cm), weights (from 400 kg to 750 kg), and breeds, which were used as leisure horses or mid-level sport horses. They were presented for loss of performance, suspected to be due to respiratory disease, or for respiratory clinical signs of varying severity typical of mild–moderate equine asthma, like occasional coughing and nasal discharge, at the Equine Clinic Hochmoor, Germany (average transportation time: 42 ± 14 min). They all underwent a respiratory examination between May 2024 and June 2025.

This study exclusively encompassed horses with an indication for examination of the respiratory tract, which was determined based on a comprehensive medical history. The data was collected as part of routine diagnostics.

Horses with severe equine asthma symptoms such as resting dyspnea and tachypnea >24 breaths/min, indicative of SEA, were excluded, along with horses with pyrexia and leukocytosis/leukopenia, as these are contraindications to bronchoalveolar lavage. In these cases, further diagnostics concerning infectious diseases followed.

### 2.2. Examination

Prior to the examination, the horses were allowed to settle for at least 30 min following their arrival at the clinic, as stressful situations result in unreliable values of the respiratory rate, respiratory type, and arterial blood gas measurement [13,19,20,21]. The acclimatization process included rest in the shade or in the stable prior to commencing the examination. A modified clinical scoring system was used to summarize the examination results, including endoscopic findings, aBGA, and BALF cytology (Table 1), resulting in a total score for each horse. The clinical scoring system was modified from the works of Ohnesorge et al. (1998) [22], which was recommended by the first Havemeyer workshop in 2001, and Barton et al. (2015) [23], which included BALF cytology, according to the latest Havemeyer suggestions concerning differentiation in mild and moderate equine asthma [7] and Beling (2022) concerning BALF cut-offs [12].

***Clinical Examination***

Each horse with anamnestic evidence of MEA underwent a complete clinical examination by an equine veterinarian (L.L., C.W., or A.B.). Examination of the respiratory system included percussion of the lungs and auscultation of the trachea, the lungs, and the heart.

The venous blood for the measurement of white blood cell count and urea was taken from the jugular vein. Hematology was completed using an automated hematology analyzer (ABAXIS VETSCAN HM5C, Griesheim, Germany). Samples were centrifuged at 3500× *g* for 2 min to obtain plasma (Hettich Zentrifugen, Kirchlengern, Germany, EBA 20, D-78532 Tuttlingen, 2002, 0114928, 6000 U/min). The concentration of urea in blood plasma was determined (FUJIFILM DRI-CHEM NX500i, Tokyo, Japan).

The blood for the arterial blood gas analysis (PaO_2_, PaCO_2_, AaDO_2_) was taken from the right carotid artery and analyzed immediately (Radiometer ABL 90 Flex, Bronshoj, Denmark). The aBGA was utilized to differentiate MEA horses from horses with SEA.

**Figure 1:**
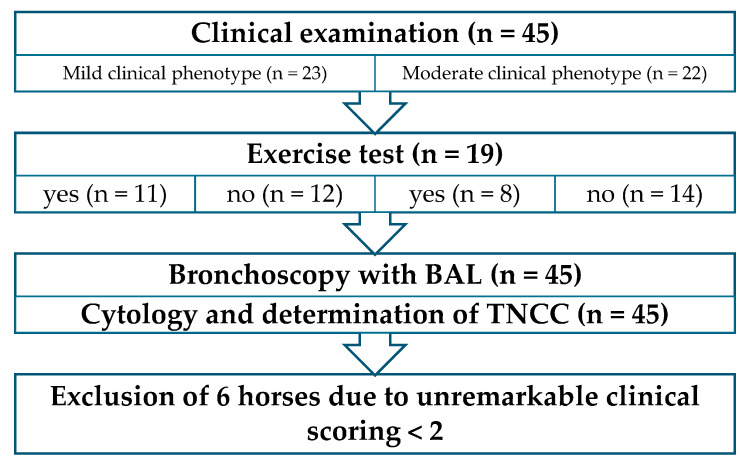
Procedure of the examination, including classification into the 2 phenotypes. **BAL**: bronchoalveolar lavage; **TNCC**: total nucleated cell count.

The detailed examination, incorporating important information from the owners, identified MEA as the leading differential diagnosis. Following this initial examination, the patients were divided into 2 groups according to the recommendation of dividing mild and moderate made by the Havemeyer Workshop in 2019 [7]: horses with respiratory symptoms (moderate clinical phenotype, *n* = 22); and horses without respiratory symptoms, but with signs of poor performance reported by the owners (mild clinical phenotype, n = 23). Horses were randomly allocated to the lunging exercise test prior to BAL, with n = 19 with and 26 without exercise prior to BAL. This resulted in 4 study groups with a presumed MEA diagnosis (Figure 1):

(1) Horses without respiratory clinical signs (mild clinical phenotype) and with exercise test (n = 11).

(2) Horses without respiratory clinical signs (mild clinical phenotype) and without exercise test (n = 12).

(3) Horses with respiratory clinical signs (moderate clinical phenotype) and exercise test (n = 8).

(4) Horses with respiratory clinical signs (moderate clinical phenotype) and without exercise test (n = 14).

***Exercise test***

The standardized lunging exercise test was conducted in an indoor round pen with irrigated equestrian sand, with a radius measuring between 8 and 10 m and a gate measuring 5 by 5 m providing access to outdoor conditions. The test consisted of a 10 min warm-up at the walk, 10 min trot, and 5 min canter, followed by a recovery phase of 10 min walking. The protocol was comparable to the study protocol of Röschmann et al., 2025 [17] and chosen to be appropriate for the population of warmbloods under investigation. Immediately after the canter phase, the heart rate and respiratory rate were assessed. The exercise test was deemed sufficient if the heart rate exceeded 100 beats per minute [17]. Horses that took longer than 15 min for the respiratory and heart rate recovery were determined to have a prolonged recovery phase. [17,24]. Thirty minutes post-exercise, the horses were sedated intravenously with 0.012 mg/kg bw detomidine (Domidine™ 10 mg/mL, Dechra Veterinary Products Deutschland GmbH, Aulendorf, Germany) and 0.025 mg/kg bw butorphanol (Torbugesic™ VET 10 mg/mL, Zoetis Deutschland GmbH, Berlin, Germany). Horses in groups 2 and 4 were sedated at the same dose immediately following the clinical exam, without performing the exercise test.

***Bronchoscopy with BAL***

For the endoscopic examination of the upper and lower airways, including BAL, a flexible endoscope of 330 cm length and 1.3 cm diameter (Karl Storz GmbH, Tuttlingen, Germany) was used.

The endoscope was maneuvered into the meatus nasi ventralis and advanced further until it reached the larynx. The nasal cavity, guttural pouch, and larynx were then evaluated. Thereafter, the endoscope was directed further into the trachea. During the bronchoscopy, the quantity and viscosity of secretions in the trachea were determined [25]. In the event of the presence of mucus, a tracheal aspirate was obtained by a sterile catheter (0.23 cm × 400 cm, WDT) for cytological analysis, and to rule out potential infectious disease. To prevent bacterial contamination of the sample, the tip (1 cm) of the catheter was sealed with sterile ultrasound gel [26]. Following the exclusion of infectious diseases on the basis of leucocyte count and rectal temperature, a bronchoalveolar lavage was conducted.

Under endoscopic control, 40 mL of local anesthetic (lidocaine hydrochloride 2%, 20 mg/mL, bela-pharm) was applied to the tracheal bifurcation by a new sterile catheter (0.23 cm × 400 cm, WDT), and bronchoalveolar lavage was performed transendoscopically following the removal of the catheter from the working channel. The endoscope was introduced into the right lung and proceeded 5–6 bifurcations into a dorsal bronchus, so it was blocked by the bronchial diameter. A total volume of 500 mL of body-warm 0.9% NaCl solution (NaCl 0.9%, 500 mL, Braun, Bella Vista, Australia) was introduced via a sterile 100 mL syringe (catheter-tip syringe without needle, 100 mL, Sol-M) through the working channel of the endoscope and immediately aspirated, which resulted in a pooled sample [27]. Microscopy was performed directly after centrifugation of the BALF at 215× *g* for 10 min [23] and following the preparation of a slide out of the pellet (Leica DM 750; Hettich Zentrifugen, EBA 20, D-78532 Tuttlingen, 2002, 0114928, 6000 U/min). For the slide preparation, a drop of the pellet mixed with 0.5 mL of BALF was placed near one end of a microscope slide. Using the edge of a second slide, the drop was spread across the surface at a 30–45° angle. Following air-drying, the smear was stained with Diff-Quick [28]. The maximum time between BALF collection, centrifugation, and preparation of the smear was 20 min each. The clinical and endoscopic findings were recorded by experienced veterinarians (L.L., C.W., and A.B.). The cytological evaluation was carried out later on, independent of the clinical and endoscopic examination, by an experienced examiner (A.B.).

All horses were admitted to the hospital for one night. Rectal temperature was recorded at the time of the procedure and subsequently in the evening and the following morning. The horses were discharged on the following day, and cases with EA received aftercare instructions for medical treatment, environmental improvement, and exercise management.

***Determination of Total Nucleated Cell Count in BALF and epithelial lining fluid (ELF)***

The total nucleated cell count in BALF was measured using an automated hematology analyzer (ABAXIS VETSCAN HM5C, Griesheim, Germany). In order to ascertain the reliability of the automated hematology analyzer in measuring the total nucleated cell count (TNCC) in BALF, the first 10 samples were also measured by a hemocytometer [28]. Therefore, the results from the automated hematology analyzer were utilized in this study. The total nucleated cell count in ELF was calculated by measuring urea in blood plasma and BALF using the “FUJIFILM DRI-CHEM NX500i, Tokyo, Japan”. The calculation was as follows: ELF (mL) = [total amount of urea in BALF recovered (mg)]/[concentration of urea in plasma (mg/mL)] [29]. With the knowledge of the recovered volume of BALF, the total nucleated cell count in BALF (hemocytometer), and the urea levels in both the blood plasma and BALF, it was possible to calculate the absolute cell count in ELF, and thereby the total nucleated cell count in ELF, in cells/µL. Urea diffuses through many compartments of the body, including the lungs. This assumption renders the effect of dilution irrelevant. The physiological cell count in ELF is typically less than 15,500–21,700 cells/µL [30]. A comparative analysis of the TNCC in ELF and BALF was conducted to assess the diagnostic value and limitations of ELF.

### 2.3. Cytological EA Diagnostic Criteria

In this study, horses were cytologically classified as mild–moderate if the BALF cytology showed >10% neutrophil granulocytes and/or >5% eosinophil granulocytes and/or >5% mast cells. Horses with >25% neutrophils in BALF cytology were cytologically categorized as severe EA. Supplementary tracheal aspirate (TA) samples were obtained in all but one horse (due to inadequate initial volumes). Theses samples were analyzed cytologically. Horses with physiological BALF cytology but increased neutrophil counts in the TA were cytologically diagnosed with EA in remission. Semi-quantitative assessment of the neutrophil count in the TA was performed and classed from − to ++++ (Table 2), based on visual assessment of 10 straight lines across the width of the slide [31,32,33]. Based on cytology, the horses were grouped into the following diagnoses: physiological, EA in remission, MEA, and SEA. A total of 1500 cells—500 cells at three distinct points on the slide—were morphologically determined by microscopy at 50× magnification [1].

**Statistical analysis**

The clinical data were documented in a digital patient documentation system (easyVET™, VetZ Gmbh, Isernhagen, Germany) and Microsoft Excel™. Statistical analysis, descriptive evaluation of the data, and chart creation were performed using the IBM SPSS Statistics 29.0.2.0 program. The initial phase involved descriptive statistics, including neutrophils, eosinophils, mast cells, lymphocytes, macrophages, TNCC in BALF, and TNCC in ELF, depending on phenotype (mild and moderate) and exercise test prior to BAL. The descriptive statistics and statistical tests were carried out partially for all 4 study groups individually, and partially separately, to determine differences in phenotypes and between the groups with or without an exercise test prior to BAL. Data were tested for normality using visual inspection of the histogram and the quantile–quantile plots, as well as descriptive parameters.

Associations between cytological diagnoses and clinical phenotype were analyzed using chi-squared tests or—if at least 25% of the cells had expected values < 5—Fisher’s exact test. Initially, all cytological diagnoses were included (physiological, EA in remission, MEA, and SEA). Subsequently, “physiological” and “EA in remission” were combined to “no EA diagnosis by BALF cytology”, and “MEA” and “SEA” were combined to “EA diagnosis by BALF cytology”, to obtain larger supergroups. Patients with missing data were excluded from the individual analyses. Differences concerning BALF cell composition between exercise-stressed and unstressed horses were investigated using the Mann–Whitney U-test.

Linear regression analysis was performed for neutrophils, TNCC in BALF, and TNCC in ELF. The influencing variable was the neutrophil count (in percent) in BALF, and the target value was TNCC in BALF and TNCC in ELF, respectively. In the third analysis, the influencing variable was TNCC in BALF and the target value was TNCC in ELF. Model diagnostics included visual inspection of normality and homoscedasticity of residuals.

A value of *p* < 0.05 was considered statistically significant.

## 3. Results

### 3.1. EA Diagnoses According to the Composition of BALF Cytology

Twenty-three horses suffered from poor performance, the typical clinical sign of mild equine asthma (six were excluded later). Twenty-two horses showed low-grade respiratory clinical signs such as coughing, nasal discharge, and increased respiratory effort after exercise, which are typical of moderate equine asthma. This led to a final study population of 39 horses (age 10.7 ± 4.8 years; bodyweight 534.2 ± 167.5 kg; height 168 ± 4.8 cm). In the present study, the majority of cases were diagnosed with neutrophilic EA (n = 24). The eosinophilic MEA subtype was observed in only three patients. There were no cases of mast cell-associated MEA. Out of 45 horses with a presumed MEA diagnosis evaluated, 6 with performance insufficiency did not exhibit any significant clinical or cytological findings in the TA or BALF and had a total score of less than 2. Three of these six horses were part of the SLET group (ID (identification number) 6, 38, 43) (Supplementary Table S1), while the others were not (ID 33, 37, 44) (Supplementary Table S2). Consequently, the presumed MEA diagnosis could not be confirmed in any of these six horses. As a result, these six horses were excluded from the statistical analysis, which yielded the following findings: By BALF cytology, three of eight exercise-stressed asthmatic horses with the mild clinical phenotype were cytologically diagnosed with SEA (37.5%) and five of them with MEA (62.5%). Two of nine asthmatic horses with the mild clinical phenotype and without exercise prior to BAL were cytologically diagnosed with MEA by BALF cytology (22.2%), and six with EA in remission by tracheal aspirate (66.7%), where the BALF cytology was physiological. One horse (ID 36; 11.1%) was classified as cytologically physiological according to the reference values utilized for BALF cytology and showed physiological TA cytology. However, this horse was included in the statistical analysis due to its neutrophil ratio of 5%.

One of eight exercise-stressed asthmatic horses with the moderate clinical phenotype was cytologically diagnosed with MEA by BALF cytology (12.5%). The other seven were either cytologically diagnosed as EA in remission by TA (n = 4/8 = 50.0%) or as physiological (n = 3/8 = 37.5%). Two of fourteen asthmatic horses with the moderate clinical phenotype and without the exercise test were cytologically diagnosed with SEA (14.3%), six with MEA (42.9%), five with EA in remission by TA (35.7%), and one as physiological (7.1%) (Table 3 and Table 4).

The results of the BALF cytology and total scores including all 45 horses are presented in tabular form (Supplementary Tables S1–S4). The median BALF recovery rate was 48.2% (interquartile range (IQR): 22–70%), and no horses had to be excluded due to a low recovery rate (<20%). Statistical analysis including the 39 determined study participants showed a median neutrophil proportion in BALF cytology of 21.0% (IQR: 18.0–51.0%) for the horses in the exercise group with the mild clinical phenotype, and 4.0% (IQR: 1.5–8.0%) for those without exercise prior to BAL. The median neutrophil proportion in the BALF cytology of horses with the moderate clinical phenotype in the exercise group was 1.5% (IQR: 0.0–4.3%), while in the non-exercise group it was 11.5% (4.5–19.5%).

**Figure 2:**
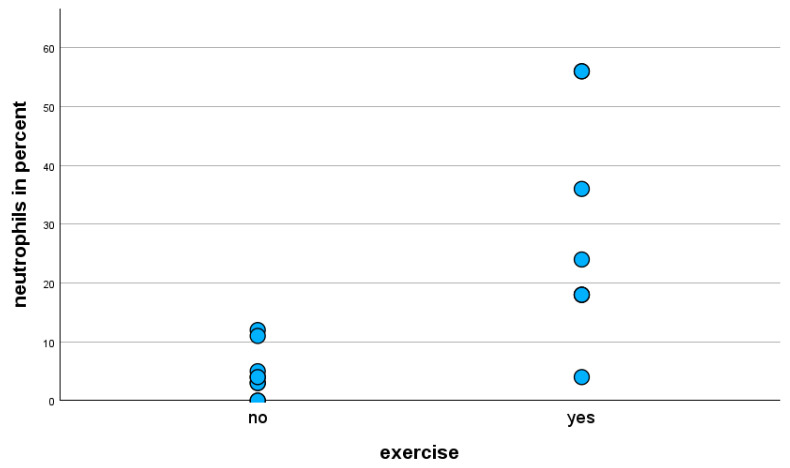
Percentage of neutrophils in BALF cytology of horses with mild clinical phenotype, with and without exercise prior to BAL.

The statistical analysis (Mann–Whitney U-test) of the BALF cell composition of exclusively horses with the mild clinical phenotype showed that exercise-stressed horses have a statistically significantly (*p* < 0.001) higher proportion of neutrophils in BALF cytology compared to unstressed horses (Figure 2). The proportion of lymphocytes in the BALF cytology of exercise-stressed horses with a mild clinical phenotype was statistically significantly lower (*p* < 0.001) than in unstressed horses.

**Figure 3:**
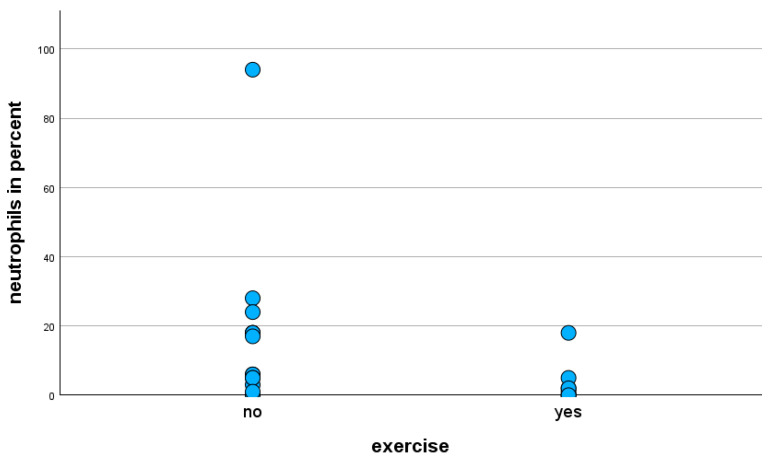
Percentage of neutrophils in BALF cytology of horses with moderate clinical phenotype, with and without exercise prior to BAL.

The Mann–Whitney U-test of exercise-stressed horses only with the moderate clinical phenotype revealed a statistically significant decrease (*p* = 0.013) in the percentages of neutrophils in BALF cytology (Figure 3).

The chi-squared test showed a statistically significantly higher prevalence of cytological MEA and SEA diagnoses, compared to a physiological finding or EA in remission (diagnosed by TA, with BALF cytology physiological) (*p* = 0.005), in the exercise-stressed horses exhibiting mild clinical phenotypes. In contrast, the exercise-stressed horses with a moderate clinical phenotype exhibited a greater frequency of physiological findings and EA in remission by TA cytology, with a lower prevalence of MEA and SEA diagnoses by BALF cytology.

To streamline the analysis, the cytological diagnoses were consolidated as previously outlined into two categories: “EA diagnosis by BALF cytology” (MEA and SEA diagnosis) and “no EA diagnosis by BALF cytology” (physiological by TA and BALF cytology or EA in remission by TA cytology). A statistically significant (*p* < 0.001) association between exercise prior to BAL and BALF cytology could be determined, to the extent that exercise-stressed horses with mild clinical phenotypes were more frequently diagnosed with EA by BALF cytology in contrast to unstressed horses, while exercise-stressed horses with moderate clinical phenotypes were less frequently diagnosed with EA by BALF cytology.

In the present study, the risk of the exercise-stressed horses with mild clinical phenotypes to affirm the presumed asthma diagnosis by BALF cytology was eight times higher compared to the exercise-stressed horses with moderate clinical phenotypes (OR = 8.00, CI = 1.28–50.04, *p* < 0.001).

If the study population is divided into an exercise group and a non-exercise group, regardless of clinical phenotype, the only significant association between exercise and BALF cytology is a lower proportion of lymphocytes in BALF cytology in the exercise group (*p* = 0.035). In detail, there is no significant association between exercise and the percentages of neutrophils (*p* = 0.767), eosinophils (*p* = 0.263), mast cells (*p* = 1.000), and macrophages (*p* = 0.217) in BALF cytology, when evaluating mild and moderate cases together.

### 3.2. TNCC in BALF

The TNCC in BALF was statistically significantly (*p* = 0.035) lower in the exercise group of equine asthmatics with moderate clinical phenotypes compared to the non-exercise group (Mann–Whitney U-test).

The median TNCC in the BALF of asthmatic horses with mild clinical phenotypes was 260.0 cells/µL (IQR: 220.0–390.0 cells/µL), whereas the median TNCC in ELF was 3062.0 cells/µL (IQR: 2740.5–5020.0 cells/µL). In horses with moderate clinical phenotypes, the median TNCC in BALF was 225.0 cells/µL (IQR: 127.5–317.5 cells/µL), and the median TNCC in ELF was 2908.0 cells/µL (IQR: 1428.5–4137.8 cells/µL) (Table 5).

Linear regression analysis including all study participants demonstrated that the percentage of neutrophils in BALF cytology exerts a statistically significant influence on TNCC in BALF (*p* < 0.001) and TNCC in ELF (*p* = 0.020). A one-percentage-point increase in the proportions of neutrophils in BALF was associated with an increase in TNCC in BALF by 4.197 cells/µL (95% confidence interval: 1.889–6.506), and with an increase in TNCC in ELF by 59.160 cells/µL (95% confidence interval: 10.026–108.295).

**Figure 4:**
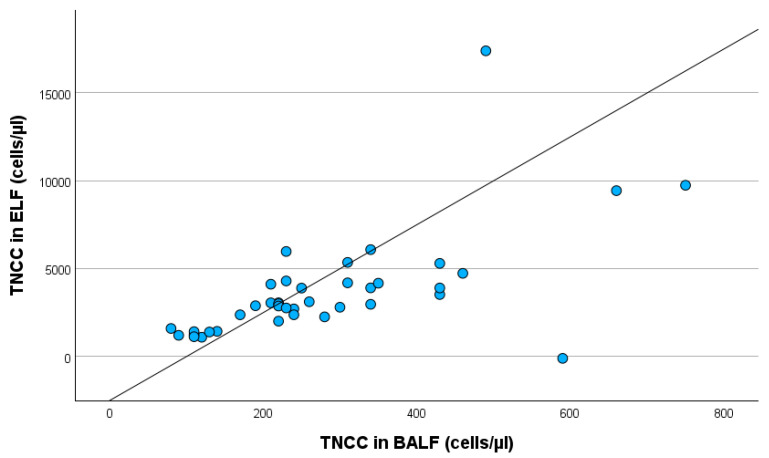
Linear regression with TNCC in BALF and TNCC in ELF, including all study participants. TNCC in BALF: total nucleated cell count in bronchoalveolar lavage fluid; TNCC in ELF: total nucleated cell count in epithelial lining fluid.

Additionally, TNCC in BALF influences TNCC in ELF statistically significantly (*p* < 0.001) (Figure 4).

An increase in TNCC in BALF by 1 cell/µL was associated with an increase in TNCC in ELF by 12.113 cells/µL (95% confidence interval: 6.974–17.252).

## 4. Discussion

This study showed that SLETs might be a suitable method to confirm the presumed diagnosis of mild EA, with a higher neutrophil ratio in BALF cytology than without exercise. The advantage of an early MEA diagnosis is to enable the initiation of treatment prior to the irreversible remodeling that might occur later on. Nevertheless, it is not yet possible to rule out the possibility that horses affected by MEA are also subject to irreversible remodeling of their bronchial smooth muscle [11].

A prolonged respiratory rate recovery after an SLET correlates with a diagnosis of EA [17]. To the best of the authors’ knowledge, the present study is the first to examine the influence of short-term exercise on BALF cytology, which is an important and practicable method for diagnosing MEA in equine practice [1,34,35]. Pulmonary function tests are the reference standard for diagnosing inflammatory airway disease [13,36,37]. Nevertheless, they are complicated to perform in patients who are not actively cooperative, like children or horses [36]. The objective was to enhance the diagnostic efficacy in terms of practicality, risk reduction, and financial viability, for which pulmonary function tests may not be the best choice.

The present test protocol—which involves 10 min of walk, followed by 10 min of trot, 5 min of canter, and finally, 10 min of walk—has been adapted to suit a range of performance levels in leisure horses and mid-level sport horses, is widely available in routine equine practice, and was recently used in a very similar way [17]. A multitude of exercise protocols have been documented in the literature. The majority of these protocols have been developed for racehorses [38]. A notable advantage of the lunging test over the treadmill test is that the horse is capable of adjusting its speed of trot and canter according to its specific condition [39]. This adaptability ensures a more precise and customized approach to the exercise test, aligning it with the specific demands of the current training condition. A comparative analysis of the effects of either lunging or treadmill on heart rate and cardiac sonography revealed no significant disparities [40]. The speed of the trot and canter can be adjusted on the lunge to ensure that the workload is adapted to the individual horse, facilitating comparable results. While an exercise test on the treadmill would offer greater standardization, it would place a greater strain on horses with a lower performance level than on horses with a higher performance level, and vice versa [39].

The current literature indicates that the respiratory tract reacts to short-term exercise. An increase in mucociliary transport and epithelial permeability after exercise leads to an elevated number of neutrophils in the tracheal aspirate. This phenomenon can be attributed to epithelial damage caused by elevated respiratory mechanics [41,42]. Epithelial damage affects the entire airway epithelium, including the alveolar epithelium, leading to a leakage of inflammatory cells and mediators into the lumen, while mucociliary transport increases [13]. In a study of asymptomatic racehorses, elevated TNCC in BALF (>530/µL) was detected after a standardized exercise. In contrast, neutrophil and eosinophil counts were not elevated [12]. Another study found an increased %PMNs in healthy horses following exercise, but no significant effect in asthmatic horses [14]. Therefore, the challenge is to determine how BALF cytology changes following short-term exercise in asthmatic horses compared to healthy ones. If exercise also changes the BALF cytology in healthy horses, there is a risk of false-positive misdiagnosis. In the present study, BALF cytology was within normal limits in twenty patients, seven of whom were in the exercise group. Therefore, SLET did not inherently lead to a cytological asthma diagnosis by BALF cytology. We cannot determine whether %PMN would have been even lower without the exercise in these patients. Nonetheless, they did not reach the threshold of >10% neutrophils and/or >5% eosinophils and/or >5% mast cells, which would have led to a cytological diagnosis of MEA. Fifteen of these twenty horses were diagnosed through TA cytology, suggesting that BALF cytology can be unremarkable in mild-to-moderate cases. An explanation for this might be that the horses were presented shortly after exacerbation, when their BALF cytology was within normal limits already but their TA cytology still showed inflammatory changes. The exacerbation in SEA is marked by a progression of symptoms that leads to dyspnea at rest. There is currently limited knowledge about the pathology, immunology, and disease progression of MEA. There are indications of a progressive course of MEA, which can develop into SEA [6]. Given EA’s high degree of variability and environmental sensitivity, the transition is expected to be seamless. From this perspective, it appears reasonable that MEA does not cause an increasingly strong inflammatory reaction in a straight line from the outset but, rather, progresses in a wave-like manner similar to SEA. The inflammatory reaction may vary in strength, depending on factors such as dust exposure, feeding practices, and weather conditions. It is possible that these phases cannot be distinguished clinically in MEA, but rather by the history of the worse phases in training reported by the owners. These phases are not currently referred to as exacerbation and remission in MEA, as MEA is not characterized by resting dyspnea. Nevertheless, the authors have decided to retain the term “EA in remission,” as this seems to be the most appropriate term for this group.

Previous studies have demonstrated an inadequate correlation between TA and BALF cytology [43,44]. It is important to note that a precise determination of the percentage of inflammatory cells in the TA does not reflect the cell pattern in the lungs. This is due to the fact that the TA is distributed over the slide in a very inhomogeneous manner and is a mixture of the secretion of the lower airways, saliva, and tracheal secretion. As TA cytology is a semi-quantitative examination, the combination of the various diagnostic steps is of great importance [31,45,46], and it may represent an additional piece of information.

The consensus statement indicates that the substantial impact of external factors on the BALF cytology of MEA horses does not permit the establishment of precise reference values. The reference values for MEA that were used in the present study are the currently accepted ones. A discrepancy exists between the reference values established for healthy horses with <5% neutrophils and those utilized for MEA with >10% neutrophils. Particularly in this grey zone, BALF cytology results should be interpreted together with all other examination results, given the dependence on technique, environment, and the horse’s daily condition [1]. In such cases, the use of TA cytology may support the MEA diagnosis and may play a more significant role in mild-to-moderate asthmatics than previously thought.

This study demonstrated a discernible distinction when comparing the prevalence of cytological asthma diagnoses by BALF cytology among horses with mild versus moderate clinical phenotypes. Exercise-stressed horses with mild clinical phenotypes exhibited a significantly higher frequency of cytological asthma diagnoses compared to those with moderate clinical phenotypes. This increased number of affirmations of the presumed asthma diagnoses can be attributed to the finding that exercise-stressed horses with mild clinical phenotypes exhibited a statistically significant elevation of neutrophils in BALF cytology compared to unstressed horses. Against the authors’ expectation, exercise-stressed horses with moderate clinical phenotypes showed statistically significantly lower neutrophil levels in BALF cytology. Eosinophils revealed no statistically significant effect; only three horses showed the eosinophilic subtype. Another notable finding was the lymphocyte ratio, which was statistically significantly lower in the exercise group regardless of clinical phenotype, as well as in mild clinical phenotypes only. Although this was a statistically significant finding, it was considered to be of low significance for the characterization of respiratory disease in horses, as it is not associated with a specific disease. Conversely, an increase in the proportion of neutrophils in BALF cytology is associated with a concomitant decrease in the proportion of another cell fraction.

The respiratory tract of horses with mild clinical phenotypes appears to be more responsive to exercise than that of those with moderate clinical phenotypes. This phenomenon is comparable to findings of the study by Benamou [14], in which the healthy horses showed an increased proportion of neutrophils in BALF cytology following exercise, but no significant effect was found in the horses with SEA following exercise. That study was conducted in 1999, so it is possible that the horses classified as “clinically healthy” could have been mild asthmatics with exercise intolerance as the only clinical sign. It has been suggested that horses exhibiting poor performance be classified as mild clinical phenotypes, and those with low-grade respiratory signs as moderate clinical phenotypes (Havemeyer in 2019). Based on this consideration, the study results might be comparable. A potential explanation for this finding might be a difference in immune response between the mild and moderate phenotypes. To the best of the authors’ knowledge, there are only studies examining differences in immune response concerning the mild–moderate or severe phenotype. The differentiation between mild and moderate is not commonly performed yet. Horses with MEA have increased mRNA expression of TNF alpha and IFN gamma [47,48]. Horses with SEA show increased mRNA expression of TNF alpha, IL-1beta, IL-4, IL-8, IL-10, and IL-17 [49,50,51,52]. A discrepancy in the immune response between mild and moderate phenotypes could lead to a different neutrophil response, as seen in BALF cytology. Assuming that EA might progress from a mild–moderate to a severe state, it appears paradoxical that horses with mild clinical phenotypes exhibit a more pronounced cytological reaction to the exercise test compared to those with moderate clinical phenotypes. For the authors, it seems possible that the lungs of asthmatic horses with mild phenotypes exhibit heightened reactivity in comparison to those of horses with moderate phenotypes. The onset of the disease in horses with a mild phenotype might be more recent compared to that of moderate asthmatics, which could explain an increased immune response, especially following the exercise test. However, due to a paucity of the literature on this subject, these are merely assumptions. Instead, the question arises as to why moderate asthmatics do not react to SLETs in the same way as mild asthmatics, which for the same reason cannot be conclusively clarified. The severity of the disease could be associated with its chronicity if a progressive course is assumed. If SEA may develop from MEA, the moderate form may also develop from the mild. Based on the results of the present study, it appears that exercise prior to BAL may be beneficial for diagnosing mild but disadvantageous for diagnosing moderate asthma.

In this study, the clinical classification according to anamnesis and symptoms did not significantly correlate with the cytological diagnosis of MEA or SEA. After the SLET, %PMN was often higher than expected. Some cases that were classified as mild or moderate by history, clinical examination, and aBGA exceeded the reference values for SEA in BALF cytology. This outcome confirms the authors’ hypothesis and supports the use of overall scoring systems, as already suggested by the first Havemeyer workshop in 2002. However, cytology is of paramount importance, as it reflects the current inflammatory status in the lungs, in contrast to the clinical classification, whereby the exercise test must be considered an amplifying factor when interpreting all results of the examination. Clinical examination may be indicative of equine asthma in SEA and asthma exacerbation, but it is often unremarkable in MEA or remission. In horses presenting with poor performance, other etiologies need to be ruled out. In addition, both cytology and clinical signs can vary greatly depending on external influences such as climate, hay quality, and dust exposure [53]. Therefore, apart from the clinical examination by the veterinarian, a comprehensive anamnesis is essential to correctly assess the clinical phenotype with the information provided by the owner. For less misunderstanding, the authors propose the utilization of the terms “clinical phenotype” and “cytological phenotype” when only referring to the clinical or cytological phenotype.

A deterioration in symptoms, manifesting as a decline in performance or intermittent coughing in response to exposure to dust, substandard hay and straw quality, and the sweeping of the dry stable alley, serves as significant evidence of EA. A combination of medical history, clinical examination including aBGA, bronchoscopy with mucus scoring [25], and cytology appears to be essential for diagnostics, as has previously been suggested in various scoring systems [7,54,55]. Prior to the administration of SLETs and BAL, the presumed diagnosis of mild-to-moderate equine asthma was determined based on a comprehensive review of the medical history and a clinical examination. Upon completion of all examination procedures, including SLET and BALF cytology, this diagnosis was subsequently validated to the evaluation and interpretation of all examination results in 39 out of 45 cases. Cytological differentiation between the two EA phenotypes—mild and moderate—has not yet been clearly defined. Beling et al. [12] propose a classification system that categorizes neutrophil percentages into mild (5–10%), moderate (11–25%), and severe (>25%), which was included in the modified scoring system used for the present study. It is crucial to note that this classification should not be applied in the absence of a comprehensive clinical evaluation, as neutrophil percentages of >5% in BALF cytology have been observed in healthy horses exposed to a dusty environment [1,56,57,58]. The present study demonstrated that horses with mild clinical signs exhibited neutrophil percentages in BALF cytology ranging from 18% to 56% following the SLET (Supplementary Table S1). High %PMN values are not necessarily associated with severe symptoms. However, in horses kept and fed in a low-dust environment, a 5% neutrophil percentage in BALF cytology may be considered suspicious for a MEA diagnosis [1,56].

In contrast to the authors’ expectations, the TNCC in BALF was statistically significantly decreased in the exercise group of equine asthmatics with a moderate clinical phenotype, compared to the non-exercise group, while another study demonstrated a higher TNCC in BALF following exercise [12]. The TNCC in BALF was generally low in the present study. According to Beling et al., an increase in TNCC in BALF > 530 cells/µL can be used as a threshold for EA diagnosis [12]. The BAL in the present study was carried out with 500 mL of sterile saline using 100 mL syringes. It is possible that the aspiration pressure levels attained in the study by Beling et al. exceeded those of the present study. This discrepancy could result in heightened epithelial damage, consequently leading to elevated levels of TNCC in BALF. Consequently, the TNCC in BALF provides no additional diagnostic value, at least not when BALF is aspirated by hand.

This study’s limitations include the small sample size and the absence of a prospective control group consisting of completely healthy horses. Nonetheless, a total of six horses (three with an exercise test prior to BAL and three without) were examined due to a history of performance insufficiency and were cytologically unremarkable, most likely having a different cause of poor performance. These horses may be considered as a control group after all. The authors decided to exclude the six horses with a loss of performance and a total score < 2, which were cytologically unremarkable from the statistical analysis. This decision was made because no other evidence during the whole examination allowed for a diagnosis of MEA. One horse from the non-exercise group (ID 36) was not excluded due to a neutrophil ratio of 5% in BALF cytology even though its TA and BALF cytology were physiological by definition. It is precisely these borderline cases that are difficult to diagnose. According to the study’s results, a diagnosis of MEA in this horse may be substantiated through the implementation of an exercise test prior to BAL.

Four horses with the moderate clinical phenotype (three with exercise test, one without) could not be diagnosed cytologically, neither by TA cytology nor by BALF cytology, and were probably in complete disease remission. As the clinical examination and medical history of these four moderate cases clearly indicated EA, they were not excluded from the study despite their unremarkable cytology. Despite an SLET prior to BAL in three of the four horses, cytology could not provide an EA diagnosis, possibly due to the mild respiratory signs and favorable environmental conditions on the day of the examination. The differences in the inflammatory response have already been discussed. The results of the total scores demonstrate that including the 39 horses with a mild–moderate clinical phenotype (total score 2–6) and excluding the 6 healthy horses (total score < 2) is a valid approach. In this study, it is not possible to differentiate between mild and moderate based on the total score. This is likely due to the classification of horses into an exercise and a non-exercise group, which provides a more pronounced cytological reaction in mild, exercise-stressed asthmatics. It seems to be effective in differentiating between mild–moderate asthmatics and healthy horses, whereas it is ineffective in differentiating between mild and moderate asthmatics in the present study. In conclusion, a comprehensive approach that incorporates medical history, clinical examination, bronchoscopy with mucus scoring, and cytology is essential to confirm the presumed diagnosis. Nevertheless, it remains difficult to diagnose horses in disease remission, as owners often do not wish for a natural challenge test prior to the diagnostic procedure.

The small sample size and study design restrict the generalizability of the findings. With only a limited number of participants, the results may not accurately represent the broader population, thereby affecting the external validity of this study. Additionally, small sample sizes increase the risk of Type II errors, where true effects may go undetected due to insufficient statistical power. This aspect is of minor consequence for this study, as we obtained significant results despite the small study population. This limitation also raises concerns about the potential overestimation of effect sizes, as smaller studies often report larger effects than are actually present. These aspects must be considered in the interpretation of the results, and SLETs, administered prior to bronchoscopy with BAL, should be regarded as an additional diagnostic step that “potentially” exhibits higher sensitivity for a mild asthma diagnosis due to a higher neutrophil proportion in BALF cytology than without an SLET.

The authors opted for the between-subjects design with two independent groups, meaning not examining the same horse before and after exercise on the same day, despite the apparent greater significance of a before-and-after test. The difficulty lies in the lack of medical indication and animal welfare for performing two BALs. Additionally, the second BAL would need to be performed in the same bronchus to ensure an identical initial situation, because differences in the cytological composition of the right and left lungs are possible [59]. However, a second BAL on the same side of the lung could be performed no earlier than 72 h following the first BAL, because an inflammatory reaction would have occurred in the sampled side of the lung as a result of the first BAL, which would be diagnostically inaccurate [60].

Additionally, there were various factors that may have influenced the examination results [53]. It is crucial to acknowledge the wide variety of EA characteristics. The results of the clinical examination on the day of examination were influenced by environmental factors such as temperature, humidity, dust exposure, and the individual’s disease status [7]. The different durations of transport to the clinic may have influenced the BALF cytology, although the effect was mitigated by the randomized allocation to the study groups. Due to the collection of results over a period of nine months, a variety of different environmental conditions were met for all included horses assessed at different times of the year.

## 5. Conclusions

The implementation of a standardized lunging exercise test appears to enhance a more definite diagnosis of mild EA, as it leads to a statistically significant increase in both neutrophil counts in BALF cytology and the number of cytologically confirmed presumed EA diagnoses by BALF cytology. In contrast, the total nucleated cell count in BALF seems to be an inaccurate tool for MEA diagnostics.

There is evidence that the BALF cytology of mild asthmatics can be unremarkable depending on environmental conditions and the patient’s condition on the day of examination. It is precisely these cases that fall just above or below the cut-off values that are the most difficult to diagnose. The aim was to improve the diagnosis of mild asthma and differentiate it from healthy horses—for example, those exposed to dust. We believe that the results of this study provide evidence that exercise before BAL might facilitate this differentiation.

Further research with a larger study population is needed to investigate the influence of exercise on BALF cytology in the mild and moderate phenotypes, as well as the differences in immune response between those two. For a future consensus statement, it could be considered to use a more comprehensible nomenclature regarding the phenotype.

## Figures and Tables

**Table 1:** Clinical scoring system, modified from Barton et al. (2015) [23], according to the latest Havemeyer suggestions (Coetil, 2020) [7] and Beling (2022) [12]. Healthy: total score < 2; mild–moderate equine asthma: total score 2–6; severe equine asthma: total score > 6. BALF cytology: bronchoalveolar lavage fluid cytology.

		Score	Max. Points
(1) Cough induction	No cough after manual compression of larynx	0	1
	Coughing during manual larynx compression	1	
	Very frequent coughing	1	
	Spontaneous coughing	1	
(2) Dyspnea at rest	Prolonged expiration	1	3
	Abdominal breathing	1	
	Sinking of intercostal area	3	
	Nostril flare	3	
	Heaves line	3	
	Anal pumping	3	
(3) Lung percussion	Three fingers	0	2
	Handbreadth	1	
	>Handbreadth	2	
(4) Lung auscultation	Rattling	2	2
	Crackle	2	
	Wheezing	2	
(5) Endoscopy	Significantly increased secretions with moderate viscosity	1	2
	Highly increased secretions with high viscosity	2	
	Thickened carina of the trachea	1	
(6) BALF cytology	Neutrophils < 5%	0	3
	Neutrophils 5–14%	1	
	Neutrophils 15–24%	2	
	Neutrophils ≥ 25%	3	
(7) Arterial blood gas analysis	AaDO_2_: 0–7 mmHg	0	2
	AaDO_2_: 7–14 mmHg	1	
	AaDO_2_: >14 mmHg	2	

**Table 2:** Scale for the semi-quantitative estimation of neutrophils in tracheal aspiration [22,32].

−	None in the entire test path
+	Isolated in the entire test path
++	Single on each line
+++	Frequently occurring on each line
++++	Dominating the cell image

**Table 3:** Cytological diagnoses of asthmatic horses with exercise test prior to bronchoscopy with BAL (16/39 horses). No.: number; EA: equine asthma; MEA: mild–moderate equine asthma; SEA: severe equine asthma; TA: tracheal aspirate; BALF: bronchoalveolar lavage fluid.

Clinical Phenotype	No. of Horses with Physiological Cytological Diagnosis	No. of Horses with EA in Remission by TA	No. of Horses with MEA (by BALF)	No. of Horses with SEA (by BALF)
Mild	0/8 (0.0%)	0/8 (0.0%)	5/8 (62.5%)	3/8 (37.5%)
Moderate	3/8 (37.5%)	4/8 (50.0%)	1/8 (12.5%)	0/8 (0.0%)

**Table 4:** Cytological diagnosis of asthmatic horses without exercise test prior to bronchoscopy with BAL (23/39 horses). No.: number; EA: equine asthma; MEA: mild–moderate equine asthma; SEA: severe equine asthma; TA: tracheal aspirate; BALF: bronchoalveolar lavage fluid.

Clinical Phenotype	No. of Horses with Physiological Cytological Diagnosis	No. of Horses with EA in Remission by TA	No. of Horses with MEA (by BALF)	No. of Horses with SEA (by BALF)
Mild	1/9 (11.1%)	6/9 (66.7%)	2/9 (22.2%)	0/9 (0.0%)
Moderate	1/14 (7.1%)	5/14 (35.7%)	6/14 (42.9%)	2/14 (14.3%)

**Table 5:** Median TNCC in BALF and median TNCC in ELF, with the interquartile range depending on clinical phenotype. TNCC in BALF: total nucleated cell count in bronchoalveolar lavage fluid; TNCC in ELF: total nucleated cell count in epithelial lining fluid; IQR: interquartile range.

Clinical Phenotype	Median TNCC in BALF (Cells/µL)	Median TNCC in in ELF (Cells/µL)
Mild	260.0 (IQR: 220.0–390.0)	3062.0 (IQR: 2740.5–5020.0)
Moderate	225.0 (IQR: 127.5–317.5)	2908.0 (IQR: 1428.5–4137.8)

## Data Availability

Data are contained within the article or Supplementary Materials; further inquiries can be directed to the corresponding author.

## Supplementary Materials

The following supporting information can be downloaded at https://www.mdpi.com/article/10.3390/ani15162428/s1: Table S1: BALF cytology and total scores of horses with mild clinical phenotypes with SLET prior to BAL; IDs 6, 38, and 43 were excluded from the statistical analysis; ID: identification number; BALF recovered: bronchoalveolar lavage fluid that was recovered. Table S2: BALF cytology and total scores of horses with mild clinical phenotypes without SLET prior to BAL; IDs 33, 37, and 44 were excluded from statistical analysis; ID: identification number; BALF recovered: bronchoalveolar lavage fluid that was recovered. Table S3: BALF cytology and total scores of horses with moderate clinical phenotypes with SLET prior to BAL; ID: identification number; BALF recovered: bronchoalveolar lavage fluid that was recovered. Table S4: BALF cytology and total scores of horses with moderate clinical phenotypes without SLET prior to BAL; ID: identification number; BALF recovered: bronchoalveolar lavage fluid that was recovered.

## References

[1] Couëtil L.L., Cardwell J.M., Gerber V., Lavoie J.-P., Léguillette R., Richard E.A. (2016). Inflammatory Airway Disease of Horses—Revised Consensus Statement. J. Vet. Intern. Med..

[2] Allen K.J., Van Erck-Westergren E., Franklin S.H. (2016). Exercise Testing in the Equine Athlete. Equine Vet. Educ..

[3] Robinson N.E., Karmaus W., Holcombe S.J., Carr E.A., Derksen F.J. (2006). Airway Inflammation in Michigan Pleasure Horses: Prevalence and Risk Factors. Equine Vet. J..

[4] Gerber V., Robinson N.E., Luethi S., Marti E., Wampfler B., Straub R. (2003). Airway Inflammation and Mucus in Two Age Groups of Asymptomatic Well-Performing Sport Horses. Equine Vet. J..

[5] Pirie R.S. (2014). Recurrent Airway Obstruction: A Review. Equine Vet. J..

[6] Bosshard S., Gerber V. (2014). Evaluation of Coughing and Nasal Discharge as Early Indicators for An Increased Risk to Develop Equine Recurrent Airway Obstruction (RAO). J. Vet. Intern. Med..

[7] Couetil L., Cardwell J.M., Leguillette R., Mazan M., Richard E., Bienzle D., Bullone M., Gerber V., Ivester K., Lavoie J.-P. (2020). Equine Asthma: Current Understanding and Future Directions. Front. Vet. Sci..

[8] Leclere M., Lavoie-Lamoureux A., Gélinas-Lymburner É., David F., Martin J.G., Lavoie J.-P. (2011). Effect of Antigenic Exposure on Airway Smooth Muscle Remodeling in an Equine Model of Chronic Asthma. Am. J. Respir. Cell Mol. Biol..

[9] Leclere M., Lavoie-Lamoureux A., Joubert P., Relave F., Setlakwe E.L., Beauchamp G., Couture C., Martin J.G., Lavoie J.-P. (2012). Corticosteroids and Antigen Avoidance Decrease Airway Smooth Muscle Mass in an Equine Asthma Model. Am. J. Respir. Cell Mol. Biol..

[10] Ceriotti S., Bullone M., Leclere M., Ferrucci F., Lavoie J.-P. (2020). Severe Asthma Is Associated with a Remodeling of the Pulmonary Arteries in Horses. PLoS ONE.

[11] Bessonnat A., Hélie P., Grimes C., Lavoie J. (2022). Airway Remodeling in Horses with Mild and Moderate Asthma. J. Vet. Intern. Med..

[12] Beling J.C.F., Santos D.M.S.A., Ferreira M.P., Silva P.C.A.R., Costa M.F.M., Lessa D.A.B. (2022). Post-exercise Endoscopic and Cytologic Diagnosis of Equine Asthma Syndrome in Asymptomatic Brazilian Pacers. Equine Vet. Educ..

[13] Lendl L., Barton A.K. (2024). Equine Asthma Diagnostics: Review of Influencing Factors and Difficulties in Diagnosing Subclinical Disease. Animals.

[14] Benamou A.E., Art T., Marlin D.J., Roberts C.A., Lekeux P. (1999). Effect of exercise on concentrations of immunoreactive endothelin in bronchoalveolar lavage fluid of normal horses and horses with chronic obstructive pulmonary disease. Equine Vet. J..

[15] Araneda O.F., Carbonell T., Tuesta M. (2016). Update on the Mechanisms of Pulmonary Inflammation and Oxidative Imbalance Induced by Exercise. Oxid. Med. Cell. Longev..

[16] Mahalingam-Dhingra A., Bedenice D., Mazan M.R. (2023). Bronchoalveolar Lavage Hemosiderosis in Lightly Active or Sedentary Horses. J. Vet. Intern. Med..

[17] Röschmann J., Naef J., Doras C., Gerber V. (2025). Respiratory Rate Recovery After Submaximal Lunging Exercise Is Delayed in Asthmatic Horses with Neutrophilic Airway Inflammation. Animals.

[18] Lendl L., Wirth C., Merle R., Barton A. Influence of a standardized lunging exercise test on BALF cytology in horses suffering from mild-moderate equine asthma. Proceedings of the Models of Lung Disease—Fraunhofer ITEM.

[19] Miller A.B., Harris P.A., Barker V.D., Adams A.A. (2021). Short-Term Transport Stress and Supplementation Alter Immune Function in Aged Horses. PLoS ONE.

[20] Art T., Lekeux P. (1995). Ventilatory and Arterial Blood Gas Tension Adjustments to Strenuous Exercise in Standardbreds. Am. J. Vet. Res..

[21] Padilla D.J., McDonough P., Kindig C.A., Erickson H.H., Poole D.C. (2004). Ventilatory Dynamics and Control of Blood Gases after Maximal Exercise in the Thoroughbred Horse. J. Appl. Physiol..

[22] Ohnesorge B., Trotschel C., Deegen E. Diagnostic value of capnography in horses with RAO. Proceedings of the 5th World Equine Vet Assoc Congress.

[23] Barton A.K., Shety T., Bondzio A., Einspanier R., Gehlen H. (2015). Metalloproteinases and Their Tissue Inhibitors in Comparison between Different Chronic Pneumopathies in the Horse. Mediat. Inflamm..

[24] Haubold A.E.M. (2006). Establishment of Reference Values for Echocardiographic Parameters of Icelandic Horses. Ph.D. Thesis.

[25] Gerber V., Straub R., Marti E., Hauptman J., Herholz C., King M., Imhof A., Tahon L., Robinson N.E. (2004). Endoscopic Scoring of Mucus Quantity and Quality: Observer and Horse Variance and Relationship to Inflammation, Mucus Viscoelasticity and Volume. Equine Vet. J..

[26] Hewson J., Viel L. (2002). Sampling, Microbiology and Cytology of the Respiratory Tract. Equine Respir. Dis..

[27] Orard M., Depecker M., Hue E., Pitel P.-H., Couroucé-Malblanc A., Richard E.A. (2016). Influence of Bronchoalveolar Lavage Volume on Cytological Profiles and Subsequent Diagnosis of Inflammatory Airway Disease in Horses. Vet. J..

[28] Couetil L.L., Thompson C.A. (2020). Airway Diagnostics. Vet. Clin. North Am. Equine Pract..

[29] Rennard S.I., Basset G., Lecossier D., O’Donnell K.M., Pinkston P., Martin P.G., Crystal R.G. (1986). Estimation of Volume of Epithelial Lining Fluid Recovered by Lavage Using Urea as Marker of Dilution. J. Appl. Physiol..

[30] McGorum B.C., Dixon P.M., Halliwell R.E.W., Irving P. (1993). Evaluation of Urea and Albumen as Endogenous Markers of Dilution of Equine Bronchoalveolar Lavage Fluid. Res. Vet. Sci..

[31] May A., Gehlen H. (2009). The examination of tracheal wash fluid and brochoalveolar lavage fluid obtains important information regarding type and severity of lung diseases. Pferdeheilkunde Equine Med..

[32] Dieckmann M.P. (1987). Zur Wirksamkeit von Ambroxolhydroxid (Mukovent^®^) Bei Lungenkranken Pferden–Klinische, Funktionelle und Zytologische Untersuchungen. Ph.D. Thesis.

[33] Robinson N.E., Chairperson W. (2001). International Workshop on Equine Chronic Airway Disease Michigan State University 16–18 June 2000. Equine Vet. J..

[34] Rossi H., Virtala A.-M., Raekallio M., Rahkonen E., Rajamäki M.M., Mykkänen A. (2018). Comparison of Tracheal Wash and Bronchoalveolar Lavage Cytology in 154 Horses With and Without Respiratory Signs in a Referral Hospital Over 2009–2015. Front. Vet. Sci..

[35] Hoffman A.M. (2008). Bronchoalveolar Lavage: Sampling Technique and Guidelines for Cytologic Preparation and Interpretation. Vet. Clin. North Am. Equine Pract..

[36] Kozłowska N., Wierzbicka M., Jasiński T., Domino M. (2022). Advances in the Diagnosis of Equine Respiratory Diseases: A Review of Novel Imaging and Functional Techniques. Animals.

[37] Robins T.-J., Bedenice D., Mazan M. (2023). A Longitudinal Analysis of Equine Asthma Presentation and Response to Treatment Using Lung Function Testing and BAL Cytology Analysis in Combination with Owner Perception. Animals.

[38] Takahashi Y., Takahashi T., Mukai K., Ebisuda Y., Ohmura H. (2024). Changes in Muscle Activation with Graded Surfaces during Canter in Thoroughbred Horses on a Treadmill. PLoS ONE.

[39] De Mare L., Boshuizen B., Plancke L., De Meeûs C., De Bruijn M., Delesalle C. (2017). Inspanningstesten Bij Paarden: Stand van Zaken En Toekomstperspectieven. Vlaams Diergeneeskd. Tijdschr..

[40] Gehlen H., Marnette S., Stadler P. (2005). Stress echcardiography in warmblood horses: Active stress induction by treadmill and longing exercise. Pferdeheilkunde Equine Med..

[41] Malikides N., Hughes K., Hodgson J. (2007). Comparison of Tracheal Aspirates before and after High-speed Treadmill Exercise in Racehorses. Aust. Vet. J..

[42] Martin B.B., Beech J., Parente E.J. (1999). Cytologic Examination of Specimens Obtained by Means of Tracheal Washes Performed before and after High-Speed Treadmill Exercise in Horses with a History of Poor Performance. J. Am. Vet. Med. Assoc..

[43] Malikides N., Hughes K., Hodgson D., Hodgson J. (2003). Comparison of Tracheal Aspirates and Bronchoalveolar Lavage in Racehorses 2. Evaluation of the Diagnostic Significance of Neutrophil Percentage. Aust. Vet. J..

[44] Derksen F.J., Brown C.M., Sonea I., Darien B.J., Robinson N.E. (1989). Comparison of Transtracheal Aspirate and Bronchoalveolar Lavage Cytology in 50 Horses with Chronic Lung Disease. Equine Vet. J..

[45] Poehlig P.J. (2024). Evaluation of the Reliability of the Cytological Examination of Tracheobronchial Secretions and Bronchoalveolar Lavage for the Diagnostics of Equine Asthma. Ph.D. Thesis.

[46] Mazan M., Pusterla N., Higgins J. (2017). Respiratory Secretions. Interpretation of Equine Laboratory Diagnostics.

[47] Lavoie J.P., Cesarini C., Lavoie-Lamoureux A., Moran K., Lutz S., Picandet V., Jean D., Marcoux M. (2011). Bronchoalveolar Lavage Fluid Cytology and Cytokine Messenger Ribonucleic Acid Expression of Racehorses with Exercise Intolerance and Lower Airway Inflammation. J. Vet. Intern. Med..

[48] Richard E.A., Depecker M., Defontis M., Leleu C., Fortier G., Pitel P.-H., Couroucé-Malblanc A. (2014). Cytokine Concentrations in Bronchoalveolar Lavage Fluid from Horses with Neutrophilic Inflammatory Airway Disease. J. Vet. Intern. Med..

[49] Laan T.T.J.M., Bull S., Pirie R., Fink-Gremmels J. (2006). The Role of Alveolar Macrophages in the Pathogenesis of Recurrent Airway Obstruction in Horses. J. Vet. Intern. Med..

[50] Wilson M.E., McCandless E.E., Olszewski M.A., Robinson N.E. (2020). Alveolar Macrophage Phenotypes in Severe Equine Asthma. Vet. J..

[51] GigueÁre S., Viel L., Lee E., MacKay R.J., Hernandez J., Franchini M. (2002). Cytokine Induction in Pulmonary Airways of Horses with Heaves and Effect of Therapy with Inhaled Fluticasone Propionate. Vet. Immunol. Immunopathol..

[52] Debrue M., Hamilton E., Joubert P., Lajoie-Kadoch S., Lavoie J.-P. (2005). Chronic Exacerbation of Equine Heaves Is Associated with an Increased Expression of Interleukin-17 mRNA in Bronchoalveolar Lavage Cells. Vet. Immunol. Immunopathol..

[53] Hotchkiss J.W., Reid S.W.J., Christley R.M. (2007). A Survey of Horse Owners in Great Britain Regarding Horses in Their Care. Part 2: Risk Factors for Recurrent Airway Obstruction. Equine Vet. J..

[54] Calzetta L., Rogliani P., Page C., Roncada P., Pistocchini E., Soggiu A., Piras C., Urbani A., Matera M.G. (2018). Clinical Effect of Corticosteroids in Asthma-affected Horses: A Quantitative Synthesis. Equine Vet. J..

[55] Gehlen H., Oey L., Rohn K., Bilzer T., Stadler P. (2008). Pulmonary Dysfunction and Skeletal Muscle Changes in Horses with RAO. J. Vet. Intern. Med..

[56] Robinson N.E. (2003). Inflammatory Airway Disease: Defining the Syndrome. Conclusions of the Havemeyer Workshop. Equine Vet. Educ..

[57] Tremblay G.M., Ferland C., Lapointe J.-M., Vrins A., Lavoie J.P., Cormier Y. (1993). Effect of Stabling on Bronchoalveolar Cells Obtained from Normal and COPD Horses. Equine Vet. J..

[58] McGORUM B.C., Dixon P.M. (1993). Evaluation of Local Endobronchial Antigen Challenges in the Investigation of Equine Chronic Obstructive Pulmonary Disease. Equine Vet. J..

[59] Depecker M., Richard E.A., Pitel P.-H., Fortier G., Leleu C., Couroucé-Malblanc A. (2014). Bronchoalveolar Lavage Fluid in Standardbred Racehorses: Influence of Unilateral/Bilateral Profiles and Cut-off Values on Lower Airway Disease Diagnosis. Vet. J..

[60] Woodrow J.S., Hopster K., Palmisano M., Payette F., Kulp J., Stefanovski D., Nolen-Walston R. (2024). Time to Resolution of Airway Inflammation Caused by Bronchoalveolar Lavage in Healthy Horses. J. Vet. Intern. Med..

