# Supplementary material for: Influence of a Standardized Lunging Exercise Test on BALF Cytology in Horses Suffering from Mild–Moderate Equine Asthma
Source: Animals (Basel). 2025 Aug 19;15(16):2428. doi: 10.3390/ani15162428 (PMC12382838; doi:10.3390/ani15162428)
Supplement: Supplementary file 1 [file animals-15-02428-s001.zip › animals-3759367-supplementary.pdf]

ID	neutrophils in %	eosinophils in %	mast cells in %	macrophages in %	lymphocytes in %	Total score	BALF recovered
1	56	0	0	18	26	4	350
2	36	2	0	48	14	4	210
3	18	5	0	52	25	4	200
4	56	1	0	19	24	5	320
5	18	0	0	72	10	3	150
7	18	0	0	65	17	5	250
34	24	0	0	54	22	4	250
35	4	5	0	60	31	2	210
6	2	0	0	56	42	1	180
38	0	0	0	70	30	1	210
43	3	0	0	46	51	1	250

Table S1. BALF cytology and total score of horses with mild clinical phenotype with SLET prior to BAL; ID 6, 38 and 43 were excluded from statistical analysis; **ID**: identification number; **BALF recovered:** Bronchoalveolar lavage fluid which was recovered

ID	neutrophils in %	eosinophils in %	mast cells in %	macrophages in %	lymphocytes in %	Total score	BALF recovered
8	3	0	0	44	53	2	320
9	4	0	0	65	31	2	260
10	0	0	0	46	54	2	300
11	3	0	0	62	35	2	300
12	12	0	0	61	27	2	240
36	5	0	0	24	72	2	120
40	11	0	0	61	28	2	210
41	4	0	0	41	55	2	320
42	0	0	0	39	61	2	320
33	0	0	0	64	37	1	280
37	0	0	0	50	50	1	150
44	0	0	0	54	46	1	280

Table S2. BALF cytology and total score of horses with mild clinical phenotype without SLET prior to BAL; ID 33, 37, 44 were excluded from statistical analysis; **ID:** identification number; **BALF recovered:** Bronchoalveolar lavage fluid which was recovered

ID	neutrophils in %	eosinophils in %	mast cells in %	macrophages in %	lymphocytes in %	Total score	BALF recovered
13	2	0	0	67	31	2	300
14	5	0	0	79	16	2	110
15	18	1	0	44	37	4	320
16	0	0	0	100	0	2	180
17	0	0	0	70	30	2	300
18	1	0	0	65	34	2	110
19	2	0	0	61	37	2	320
39	0	0	0	60	40	2	130

Table S3. BALF cytology and total score of horses with moderate clinical phenotype with SLET prior to BAL; **ID**: identification number; **BALF recovered:** Bronchoalveolar lavage fluid which was recovered

ID	neutrophils in %	eosinophils in %	mast cells in %	macrophages in %	lymphocytes in %	Total score	BALF recovered
20	18	0	0	54	28	4	180
21	6	5	0	43	46	2	300
22	0	0	0	100	0	2	110
23	18	0	0	58	24	2	320
24	94	0	0	6	0	6	190
25	3	0	0	71	26	3	320
26	6	2	0	73	19	2	180
27	5	0	0	67	28	2	270
28	5	0	0	52	43	3	190
29	28	0	0	39	33	4	300
30	1	0	0	54	45	2	180
31	18	0	0	62	20	3	250
32	24	0	0	36	40	6	190
45	17	0	0	39	44	2	220

Table S4. BALF cytology and total score of horses with moderate clinical phenotype without SLET prior to BAL; **ID**: identification number; **BALF recovered:** Bronchoalveolar lavage fluid which was recovered
